# The Prevalence of Dyslipidemia in Patients with Spinal Cord Lesion in Thailand

**DOI:** 10.1155/2012/847462

**Published:** 2012-07-10

**Authors:** Ratana Vichiansiri, Jittima Saengsuwan, Nuttaset Manimmanakorn, Sirasaporn Patpiya, Arayawichanon Preeda, Kharmwan Samerduen, Ekasit Poosiripinyo

**Affiliations:** Department of Physical Medicine and Rehabilitation, Faculty of Medicine, Khon Kaen University, Amphur Muang, Khon Kaen 40002, Thailand

## Abstract

*Objective*. To assess the prevalence of dyslipidemia in 90 patients with spinal cord lesion (SCL) of duration greater than 2 years. The study was carried out from November 2007 to September 2008. *Methods*. Clinical history, physical examination, and lipid profiles were recorded and analyzed. Dyslipidemia was assessed using guidelines from the National Cholesterol Education Project Adult Treatment Panel III (ATP III). *Results*. The prevalence of dyslipidemia in at least one lipid parameter was 76.7%. The most frequent finding was low HDL-C (58.9%). Hypertriglyceridemia, hypercholesterolemia and high LDL-C were found in 28.9%, 26.7% and 21.1% of patients, respectively. The factors sex = male and age ≥45 years were associated with high LDL-C (*P* < 0.05 and *P* < 0.01). Patients who exercised less than 30 minutes per day had associated hypercholesterolemia (*P* < 0.01), hypertriglyceridemia (*P* < 0.01), and higher LDL-C (*P* < 0.05). Patients with BMI ≥23 kg/m^2^ had associated hypercholesterolemia and high LDL-C. Age was a significant determinant of high LDL-C. BMI was the most powerful and significant determinant of hypercholesterolemia and high LDL-C. *Conclusion*. SCL patients should have a regular lipid checkup, especially those patients having the following risk factors: males, age ≥45 years, BMI ≥23 kg/m^2^ and exercise duration <30 minutes per day.

## 1. Introduction

There has been great improvement in therapeutic care for patients with spinal cord injury (SCI) but patients still have lower-than-normal life expectancy. It has been found that life expectancy of tetraplegia and paraplegia is less than in the normal population [1]. The major established cause of death is cardiovascular disease [2, 3].

Whiteneck [4] reported that cardiovascular disease accounted for nearly half of all deaths in persons with spinal cord injury of duration greater than 30 years. Lee et al. [5] found that the prevalence of asymptomatic coronary artery disease was 63.8% as determined by Thallium-201 myocardial perfusion single-photon emission-computed tomography (T201-SPECT). There are many important risk factors in developing cardiovascular disease such as a decrease in physical activity, obesity, metabolic syndrome, dyslipidemia, impaired glucose tolerance, and decreased cardiovascular fitness [6].

Dyslipidemia is one of the most important risk factors and can be modified for primary prevention in cardiovascular disease. In this study the prevalence of dyslipidemia was determined from ATP III guidelines which were developed from the National Cholesterol Education Program (NCEP) Expert panel on Detection, Evaluation, and Treatment of High Blood Cholesterol in 2001. ATP III sets guidelines for cardiovascular risk evaluation and management [7–9]. It differs from the previous ATP I-II guidelines in the emphasis of primary prevention. The new guidelines decrease the triglyceride (TG) cutoff point for hypertriglyceridemia from ≥200 mg/dL to TG ≥150 mg/dL) increase the HDL cholesterol (HDL-C) cutoff points (HDL-C < 35 to HDL-C < 40 mg/dL), and include metabolic syndrome evaluation [9–11].

Nash and Mendez [12] studied dyslipidemia in 41 paraplegic patients following ATP III guidelines and found that as many as 76% of patients had HDL-C levels less than 40 mg/dL and up to 63.4% need further treatment.

Many studies on the prevalence of dyslipidemia have been conducted in western countries, but data in Asia is still scarce. Since there are many regional differences in genetics, way of life, and diet, the present study was designed to examine the mean lipid level and prevalence of dyslipidemia among spinal cord lesion patients in Thailand.

## 2. Materials and Methods

### 2.1. Settings and Subjects

This was a cross-sectional, descriptive study. Participants involved in the study were patients with chronic SCL (duration at least 2 years) either from spinal cord injury of traumatic or nontraumatic cause and intrinsic cord problem such as myelomeningocele who were admitted for urological review to Srinagarind Hospital, located in Khon Kaen in the Northeast of Thailand, from 1 November 2007 to 30 October 2008.

This study included 90 patients (55 men and 35 women) aged 18–80 years. Patients who had a history of diagnosed dyslipidemia, as well as patients who had any condition that could alter lipid profiles were excluded from the study.

The study was conducted in accordance with the 1975 Helsinki Declaration (revised 1983), approved by the Ethics Committee of Khon Kaen University. Written informed consent was obtained from all of the subjects.

### 2.2. Specimen Collection

Serum samples were collected from subjects in the morning after 10 hours of fasting. Measurements included total cholesterol (TC), triglyceride (TG) and high-density lipoprotein cholesterol (HDL-C). Low-density lipoprotein cholesterol (LDL-C) was calculated by the following equation [13]:

(1) LDL-C  =  TC−(HDL-C  +TG5).

### 2.3. NCEP III Guidelines

The classification of dyslipidemia was based on the NCEP III Guidelines. These classify lipid profile as abnormal if, HDL-C is less than 40 mg/dL; TC is more than 200 mg/dL, or TG is more than 150 mg/dL. High LDL-C is classified based on 5 risk factors: age (men ≥45 years and women ≥55 years), cigarette smoking, hypertension or currently taking antihypertensive medication, HDL-C level, and family history of premature heart disease. In patients with 1 risk factor, the cutoff point of LDL-C is 160 mg/dL. The cutoff point is 130 mg/dL for patients with 2 or more risk factors. In patients with coronary heart disease (CHD) or CHD risk equivalent, such as diabetes or atherosclerotic disease, the cutoff point of LDL-C is 100 mg/dL. 

### 2.4. Framingham Risk Score

Framingham risk scores were calculated based on the Framingham study [14, 15]. This is widely used to classify people with risk factors into 3 distinct groups (low risk, moderate risk, and high risk), which is based on the observed risk of developing CHD or cardiac cause of death in 5,209 healthy men and women aged 30 to 62 in Framingham, MA, USA. The risks that are accounted for in the calculation are age, gender, TC, HDL-C, cigarette smoking, and systolic blood pressure or the use of antihypertensive drugs. The score calculated is further transformed into a percentage. If the percentage is less than 10%, the classification is low risk; 10–20% will be classified as intermediate risk and greater than 20% will be classified as high risk.

### 2.5. Statistical Analysis

Statistical analyses were performed on a personal computer using SPSS version 16.0 (SPSS, Inc, Chicago). The results were expressed as means and standard deviations. Chi-squared analyses and Fishers Exact tests were performed to determine factors associated with dyslipidemia and Framingham risk categories. Multiple regression analysis was used to investigate the influences of age, gender, family history of coronary artery disease, smoking, exercise duration, BMI, level of injury, completeness of lesion, duration after injury, and ambulatory level to predict dyslipidemia of any type of lipid. 

## 3. Results

After excluding 6 subjects, data from 90 subjects were analyzed: 62 paraplegics and 28 tetraplegics. Average age was 42.3 (range 18–80 years). Six of the participants were smokers, and 9 regularly consumed alcohol. Five patients had a family history of coronary artery disease.

According to the clinical history, the spinal cord lesion was complete in 51 participants (56.7%). Fifty-five (61.1%) participants were wheelchair users. The average time after spinal cord injury was 5.4 years. Thirteen patients did not exercise (Table 1).

There were 69 participants (76.7%) who had abnormality in at least one lipid profile. In the dyslipidemic group, there were 54 patients (78.3%) who had abnormal lipid profile of more than 1 type, and there were only 15 patients (21.7%) who had dyslipidemia diagnosed from only one profile.

Fifty-three participants (58.4%) had a depressed mean serum HDL-C level, leading to the average level of 39.4 mg/dl as shown in Table 2. TG, LDL-C, and TC were found abnormal in 28.9%, 21.1%, and 26.7% of patients, respectively.

### 3.1. Factors Associated with Dyslipidemia

Factors that were demonstrated to be associated with dyslipidemia are shown in Table 3. Male sex and age of more than 45 years was associated with high LDL-C (*P* < 0.05 and *P* < 0.01). An average exercise time of less than 30 minutes per day was associated with hypercholesterolemia, hypertriglyceridemia (*P* < 0.01), and higher LDL-C (*P* < 0.05). BMI equal to or greater than 23 kg/m^2^ was associated with hypercholesterolemia and high LDL-C.

This study did not demonstrate any significant correlation of the level of injury (tetraplegia or paraplegia), completeness of injury, and duration of injury together with ambulatory level of the patients with dyslipidemia from any type of lipid. 

Results of the regression analysis, investigating the influence of subject characteristics and behavioural factors on the lipid profile, are listed in Table 4. Age was a significant determinant of high LDL-C. BMI was the most powerful and significant determinant of hypercholesterolemia and high LDL-C.

### 3.2. Risks of CHD Based on Framingham Risk Score

Fourteen patients (15.6%) were in the moderate-to-high-risk group for hard CHD or cardiovascular death based on the Framingham risk score. All of them were men and aged more than 45 years. In the moderate-to-high-risk group, there were 71.4% of patients who exercised less than 30 minutes per day; in the low risk group, 57.9% of patients exercised less than 30 minutes per day. There was a higher percentage of patients who smoke in the high-risk group although the difference is not statistically significant. There was no significant difference in association with abdominal circumference, duration of SCL, level of lesion, ambulatory method, and BMI between the low- and high-risk groups (Table 5).

## 4. Discussion

Individuals with SCI were found to have higher risk of CHD at a younger age. Dyslipidemia is an important modifiable risk factor and with good prevention and control, morbidity, and mortality may be delayed for this group of patients in the future. Using the NCEP (ATP III) Guidelines, this study shows that the prevalence of dyslipidemia is high. Depression of HDL-C level accounted for major abnormalities in lipid profile. This finding was consistent with the study of Nash and Mendez who found abnormality in HDL-C levels in 31 out of 41 participants with paraplegia [12]. This study also correlates with other studies [16–20] which showed that low HDL-C is the most significant lipid alteration after SCI. The low HDL-C may be explained by lower physical activity in the SCI group; previous reports found that sedentary lifestyle leads to an accentuated state of insulin resistance, and this leads to higher triglyceride, uric acid, and lower HDL-C levels [21, 22]. Although immobilization plays a major role in HDL-C levels, other causes of lower HDL-C such as smoking, excessive alcohol intake, and high-fat diet may also contribute to low HDL-C levels [23]. There are many studies that support the proposal that HDL-C helps prevent atherosclerosis through various mechanisms such as antioxidant effects or inhibition of endothelial destruction [24, 25]. An optimum HDL-C level will need to be determined, and effective ways to help improve HDL-C levels, either by medication or exercise, should be found so as to most effectively help this group of patients [26].

A TG level of more than 150 mg/dL led to higher overall mortality and incidence of cardiovascular disease. Comparing TG levels from the study of Nash and Mendez [12] to the population in this study, it was found that there was a lower mean TG level, which may have been caused by a difference in genetics, habits, or diet.

There was a difference in the prevalence of low HDL-C and high LDL-C between genders which may have been a result of different sex hormones that influence blood lipid profiles [27, 28].

Age and BMI were shown to be associated with high LDL-C levels, and the results also correlate with a previous study comparing the factors associated with lipid profiles in active and sedentary tetraplegic patients [29]. However, compared to the study of Bauman et al. [20], which determined the effect of residual neurological deficit on lipid profiles in patients with chronic spinal cord injury and found that there was a significant inverse relationship between the degree of neurological deficit and mean serum HDL-C levels, this study did not demonstrate any significant association between level of injury, completeness of injury, or ambulatory level and dyslipidemia. When analyzed in detail, the significance of neurological level may be confounded by the effect of age as the mean age for tetraplegic patients was 38.3 years compared to the higher average age of 44.2 years in paraplegia. This might have a substantial effect considering that age accounted for a significant alteration in lipid level.

 When comparing the prevalence of dyslipidemia in SCL patients in Thailand with the study of the normal population by Pongchaiyakul et al. [30], the SCL patients showed a significant difference in the prevalence of HDL-C compared to the normal population. The prevalence of hypertriglyceridemia, hypercholesterolemia, and high LDL-C was comparable.

Interestingly, based on the Framingham risk score, all patients with moderate-to-high risk were male and more than 45 years of age; all patients in the high-risk group had low HDL-C levels. BMI and abdominal circumference were not significantly different from the low-risk group. The greater proportion of moderate-to-high-risk patients classified by exercise duration of less than 30 minutes showed a trend for the effect of exercise; lack of significance may be due to the small sample size. Most patients in the moderate-to-high-risk group (78.6%) have a duration of SCL of less than 5 years. This also agrees with the study of Dallmeijer et al., which found lower HDL-C levels within 2 years after SCI [19]. This may imply that physicians should check lipid profiles and estimate the CHD risk even shortly after SCI, especially in men who are more than 45 years of age.

After the lipid screening, the prevalence of SCL patients with at least one lipid abnormality is 76.7%. Seven patients (10.1%) had an indication for lipid-lowering medication based on NCEP III guidelines, and 62 patients (89.9%) were given advice for therapeutic lifestyle modification.

One of the limitations of this work is the cross-sectional nature of the study. We did not follow up with the patients since the onset of SCI so we do not know whether males aged more than 45 years had this risk before injury or not.

In conclusion, the high percentage of dyslipidemia found in SCL patients supports the increased risk of CHD in this group. There is an indication for mandatory annual checking of the lipid profile especially in the high-risk group: those who are male, more than 45 years of age and exercising for less than 30 minutes a day. Moreover, regular followup to advise for activity modification such as eating habits, exercise, or medication, that is, medical intervention to prevent cardiovascular disease, should be incorporated in the routine clinical followup.

## Figures and Tables

**Table 1:** General Characteristics of 90 spinal cord lesion patients (SD: standard deviation).

Characteristics	Value
Number of subjects	90
Age, years, mean (SD)	42.3 (13.7)
Male/Female, *n* (%)	55 (61.1)/35 (38.9)
Family history of coronary artery disease, *n* (%)	5 (5.6)
Duration of injury, years, mean (SD)	5.4 (4.7)
Current smoker, *n* (%)	6 (6.7)
Level, *n* (%)	
Paraplegia	28 (31.1)
Tetraplegia	62 (68.9)
Completeness, *n* (%)	
Complete	51 (56.7)
Incomplete	39 (43.3)
Ambulatory ability, *n* (%)	
Wheelchair	55 (61.1)
Walk	35 (38.9)
Exercise, *n* (%)	
No exercise	13 (14.4)
Exercise	
<30 min/day	41 (45.6)
>30 min/day	36 (40)

**Table 2:** Lipid profile values (mean (SD)).

Type	Blood level	
	mg/dL	Range
HDL-C	39.4 (10.4)	23–71
TG	130.5 (86.3)	38–572
LDL-C	111.0 (36.8)	44–196
TC	175.1 (41.3)	87–294

**Table 3:** Factors associated with dyslipidemia classified by each type of lipid.

Factors	Normal	Dyslipidemia	*P* value
HDL-C			
Male	17/24 (70.8%)	38/66 (57.6%)	0.04
Age ≥ 45 years	6/24 (25%)	25/66 (37.9%)	0.32
BMI ≥ 23 kg/m^2^	3/24 (12.5%)	13/66 (19.7%)	0.54
Exercise less than 30 min/day	13/24 (54.2%)	41/66 (62.1%)	0.21
LDL-C			
Male	16/19 (84.2%)	39/71 (54.9%)	0.02
Age ≥ 45 years	13/19 (68.4%)	18/71 (25.4%)	0.000
BMI ≥ 23 kg/m^2^	7/19 (36.8%)	9/71 (12.7%)	0.014
Exercise less than 30 min/day	16/19 (84.2%)	38/71 (53.5%)	0.015
TG			
Male	18/26 (69.2%)	37/64 (57.8%)	0.31
Age ≥ 45 years	12/26 (46.2%)	19/64 (29.7%)	0.14
BMI ≥ 23 kg/m^2^	7/26 (3.8%)	9/64 (14%)	0.15
Exercise less than 30 min/day	21/26 (81.7%)	33/64 (51.6%)	0.01
TC			
Male	16/24 (66.7%)	39/66 (59%)	0.51
Age ≥ 45 years	12/24 (50%)	19/66 (28.8%)	0.06
BMI ≥ 23 kg/m^2^	9/24 (37.5%)	7/66 (10.6%)	0.003
Exercise less than 30 min/day	20/24 (83.3%)	34/66 (51.5%)	0.006

**Table 4:** Results of multiple logistic regression analysis: effects of age and BMI on TC and LDL-C.

Dependent variables	Independent variables	Crude OR	Adjusted OR	95% CI for adjusted OR	*P* value
hypercholesterolemia	BMI < 23 kg/ m^2^	2.0	1	2.0–57.4	0.006
	≥23 kg/m^2^	20.7	10.7		

High LDL-C	Age < 45 years	1.0	1	1.3–13.9	0.015
	≥45 years	6.4	4.3		
	BMI < 23 kg/m^2^	1.0	1.0	1.2–56.7	0.034
	≥23 kg/m^2^	4.5	8.1		

**Table 5:** Factors associated with moderate to high risk based on the Framingham risk score.

Factors	Low risk (<10%)	Moderate/high risk (≥10%)	*P-*value
Male	41/76 (53.9%)	14/14 (100%)	0.001
Age ≥ 45 years	17/76 (22.3%)	14/14 (100%)	0.000
Tetraplegia	25/76 (32.8%)	3/14 (21.4%)	0.30^∗^
WC ambulation	48/76 (63.2%)	5/14 (35.7%)	0.55
Duration of SCL ≥ 5 years	23/76 (30.3%)	3/14 (21.4%)	0.75^∗^
Cigarette smoking	4/76 (5.3%)	3/14 (21.4%)	0.07^∗^
Abdominal circumference	11/76 (14.5%)	1/14 (7.1%)	0.68^∗^
BMI ≥ 23 kg/m^2^	15/76 (7.1%)	1/14 (7.1%)	0.45^∗^
Exercise less than 30 min/day	44/76 (57.9%)	10/14 (71.4%)	0.39

^∗^Fisher's exact test.
